# Graphene-Based and Surface-Enhanced Raman Spectroscopy for Monitoring the Physio-Chemical Response of Thermophilic Bacterial Spores to Low Temperatures Exposure

**DOI:** 10.3390/s20154150

**Published:** 2020-07-26

**Authors:** Carlo Camerlingo, Giuseppe Di Meo, Maria Lepore, Mikhail Lisitskiy, Annarita Poli, Marianna Portaccio, Ida Romano, Paola Di Donato

**Affiliations:** 1 CNR-SPIN, Institute for Superconductivity, Innovative Materials and Devices, 80078 Pozzuoli, Italy; carlo.camerlingo@spin.cnr.it (C.C.); mikhail.lisitskiy@spin.cnr.it (M.L.); 2 CNR-ICB, Institute of Biomolecular Chemistry, 80078 Pozzuoli, Italy; peppe_dimeo1989@hotmail.it (G.D.M.); annarita.poli@icb.cnr.it (A.P.); iromano@icb.cnr.it (I.R.); paola.didonato@icb.cnr.it (P.D.D.); 3 Dipartimento di Medicina Sperimentale, Università della Campania “L. Vanvitelli”, Via S. Maria di Costantinopoli 16, 80138 Napoli, Italy; marianna.portaccio@unicampania.it; 4 Dipartimento di Scienze e Tecnologie, Università Parthenope di Napoli, Centro Direzionale Isola C/4, 80143 Napoli, Italy

**Keywords:** thermophilic bacteria, spore germination, SERS, graphene-based pH-meter

## Abstract

Monitoring the spore life cycle is one of the main issues in several fields including environmental control, sustainable ecosystems, food security, and healthcare systems. In this framework, the study of the living organism resistance to extreme conditions like those mimicking space environments is particularly interesting. The assessment of the local change of the pH level can be extremely useful for this purpose. An optical physiometer method based on the Raman response of the graphene, which is able to locally sense pH of a fluid on a micrometric scale, has been recently proposed. Due to the presence of π-bonds at the surface, the electronic doping of graphene is determined by the external conditions and can be electrochemically controlled or altered by the contact with an acid or alkaline fluid. The doping level affects the vibrational energies of the graphene that can be monitored by conventional Raman spectroscopy. In addition, Surface-Enhanced Raman Spectroscopy (SERS) can give direct information on the biochemical changes occurring in spore components. In this work, we propose the joint use of Graphene-Based Raman Spectroscopy (GbRS) and SERS for the monitoring of the response of spores to exposure to low temperatures down to 100 K. The spores of the thermophilic bacterium *Parageobacillus thermantarcticus* isolated from an active volcano of Antarctica (Mt. Melbourne) were investigated. These spores are particularly resistant to several stressing stimuli and able to adapt to extreme conditions like low temperatures, UV irradiation, and γ-rays exposure. The results obtained showed that the joint use of GbRS and SERS represents a valuable tool for monitoring the physio-chemical response of bacterial spores upon exposure to stressing stimuli.

## 1. Introduction

**Figure 1:**
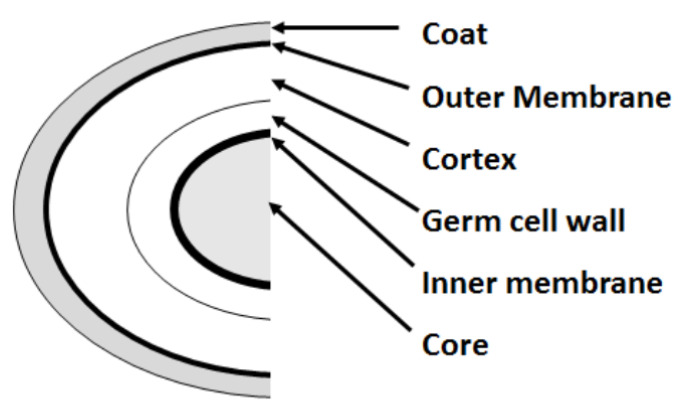
A schematic representation of the layers of a bacterial spore belonging to the *Bacillus* genus. The external coat is a protein layer that surrounds the outer lipid membrane; below this membrane, two peptidoglycan layers i.e., where the cortex and the germ cell wall coat are located. The inner membrane (in which different proteins acting as germination receptors and channels mediating the exchange of molecules and ions) surrounds the spore core where DNA and proteins are suspended in a Ca${}^{2+}$-DPA that act as a protection against harmful chemical or physical agents.

The study of biochemical changes occurring in the different stages of the Bacilli spore life cycle is of large interest from both the basic and applied point of view. Spores are dormant cells that display great resistance to several harmful chemical and physical agents. Thanks to their unique structure and chemical composition, spores can extend their life an extremely long time on a biological time scale. The underlying mechanisms constitute an intriguing challenge from the scientific point of view with potentially important clues on the applications and comprehension of fundamental questions concerning the origin of life on the Earth [1]. A schematic representation of the typical multi-layer structure of Bacilli spores is depicted in Figure 1. An external shell made of proteins (the coat) surrounds the outer membrane under which a thick layer of modified peptidoglycan, i.e., where the cortex and the germ cell wall (composed of a peptidoglycan matrix) are located. The inner membrane lays under all these structures and includes the spore core in which the DNA, ribosomes, and enzymes are suspended in a matrix of Ca${}^{2+}$-DPA (calcium dipicolinate, where DPA stands for dipicolinic acid). This substance serves as a protection from harmful external agents and is released upon the germination or as a response to chemical or physical stimuli that can activate the channels that allow the Ca${}^{2+}$-DPA to leave the core [2]. Both the inactivation and germination processes of Bacilli spores have been intensely studied for practical purposes, e.g., the development of sanitary and food industry treatments for preventing contaminations [3,4,5,6] and for more fundamental aspects, such as the search of the environmental conditions for triggering spore germination and then viable cell growth in extreme conditions like those mimicking space environments [7,8,9]. The assessment of the local change of the pH levels can be useful for the comprehension of the involved mechanisms as it provides complementary information on the substance released from spores during life cycle processes [10]. An optical pH physiometer method based on the Raman response of the graphene, which is able to locally sense the acidity of fluids on a micrometric scale, has been proposed by Paulus et al. [11]. Due to the presence of π-bonds at the surface, the electronic doping of graphene is determined by the external conditions and can be electrochemically controlled [12] or altered by the contact with an acid or alkaline fluid [11,13]. The doping level affects the vibrational energies of the graphene that can be monitored by conventional Raman spectroscopy. Additional information on the biochemical changes can also be obtained by means of Raman spectroscopy [14,15,16,17] or Surface-Enhanced Raman Spectroscopy (SERS) [18,19]. In particular, a great interest is devoted to SERS due to its high sensitivity and selectivity [20]. The joint use of Graphene-Based Raman Spectroscopy (GbRS) and SERS can represent a fruitful method to characterize and monitor the biochemical changes occurring during the different phases of spore life cycle or the effects caused by the exposure to external chemical or physical stimulus. A particularly interesting external stimulus for spores can be represented by extremely low temperatures that can mimic the space environment as the average temperature minimum (around 133 K) occurring on Mars soil along the Martian daily cycle [21].

In this work, we propose the combined use of GbRS and SERS for monitoring the changes occurring in spores due to the exposure to low temperatures down to 100 K. The former technique enabled us to obtain a pH estimation using very small quantities of samples, while SERS can be used for monitoring the response of spores to thermal stresses. For this study, we selected spores of the thermophilic bacterium *Parageobacillus thermantarcticus* isolated from an active volcano in Antarctica (Mt. Melbourne) [22]. The viable cells and spores of *Parageobacillus thermantarcticus* are particularly resistant to several stressing stimuli and able to adapt to extreme conditions like low temperatures, UV irradiation, and γ-rays exposure, as previous studies showed [14,23,24]. The ability of these microorganisms to survive in life-hostile environments is often recalled as support of the panspermia hypothesis of the extraterrestrial origin of life [1]. The assessment of resistance limits to physical agents, as low temperatures, is an essential element for speculating on the hypothesized microorganism transport across the space by meteorites. Furthermore, an insight into the spore behavior at low temperatures can be relevant for the development of inactivation processes aimed to control food conservation and safety.

## 2. Materials and Methods

### 2.1. Spore Cultivation and Collection

Spores of the bacterium *Parageobacillus thermantarcticus* (strain M1) were generated by using as the sporulating medium YNM (0.6% yeast extract, 0.3% NaCl, and 0.001% MnSO${}_{4}$, pH 5.6–6.8 in tap water). A total of 1 ml of the strain solution was inoculated in 100 ml of YNM medium (1:100 *v*/*v*) and then incubated at 60 °C in static conditions for 48 h; the sporulation process was monitored using optical microscope analysis. Both vegetative cells and spores were harvested by centrifugation at 10,000 rpm for 15 min, the resulting pellet was washed with sterile distilled water and spores were recovered by centrifugation at 4000 rpm for 15 min. The washing treatment was repeated until the spore suspension contained 99% phase-bright spores, as confirmed by phase-contrast optical microscopy analysis. The obtained spores suspension was placed in a Corning tube and stored at 4 °C until Raman spectroscopy analysis was performed.

### 2.2. Graphene-Based pH Physiometer

Graphene was employed to implement a pH physiometer following the experimental design proposed by Paulus et al. [11]. We used high quality 10×10 mm${}^{2}$ graphene substrates purchased from Graphenea (San Sebastiàn, Spain). A drop of about 1 μL of spores suspension was placed on the graphene surface and analyzed by micro-Raman spectroscopy after drying. The Raman signal was acquired using a conventional micro-Raman apparatus, constituted by a Jobin–Yvon system (Horiba, Japan) with a TriAx 180 monochromator with a spectral resolution limit of 2 cm${}^{-1}$ (grating of 1800 lines/mm). The Raman signal was excited using a 17 mW He-Ne laser and collected by a liquid nitrogen cooled Charge-Coupled Detector (CCD). The Raman signal was acquired at room temperature using a microscope objective 100 × (n.a. = 0.90) with accumulation time of 180 s. In order to investigate the changes occurring in the pH level due to thermal stress, the spore samples were exposed to low temperatures using a closed-cycle cryocooler (Lake Shore Cryotronics, Westerville, OH, USA), with temperature stability of 0.1 K. The Si substrate holding the graphene/spores sample was attached to the cryostat cold-finger using silver paint. The samples were cooled in vacuum conditions at decreasing temperatures, $T_{e}$ = 294 K, 260 K, 230 K, 200 K, 170 K, 140 K, 133 K, 120 K, and 100 K and were kept at the fixed temperature for 15 min. After this exposure time, the samples were slowly warmed up to room temperature and then the graphene spectra were acquired for the pH determination. For each temperature at less four spectra were collected in different positions of the sample area.

### 2.3. Surface-Enhanced Raman Spectroscopy

SERS was implemented by using a home-made colloid of Gold Nanoparticles (GNPs). GNPs were obtained by a chemical process based on citrate reduction of HAuCl${}_{4}$ and had a diameter of 26 nm that has been shown to give an optimal SERS response for the He-Ne laser source used in the experiments. Details on GNP preparation and characterization are available in [25] and references therein. A SERS signal enhancement of the order of $10^{3}$ was obtained for Rhodamine 6G/water solutions using a concentration as low as 5 μM [26]. For the present series of measurements, GNPs were added to a 1μL drop of the spores suspension, and slightly reshuffled by using the pipette tips. The drop was left to dry on a 1 mm thick copper substrate for almost half an hour into a laminar flow cabinet and after covered by a microscope coverslip (50 μm thick). The glass edges were sealed by epoxy glue to the copper substrate. Spore suspensions after the addition of GNPs did not show any visible change when observed by an optical microscope, but the use of GNPs allowed us to take advantage of the enhancement that SERS effect provides when the substance to probe is physically close to the GNP surface. The SERS signal was collected by means of a dedicated optical system that was employed for cryogenic in-situ measurements using the cryostat previously described for GbRS implementation. In particular, we used a 20× long-distance optical objective placed at a distance of 20 mm from the sample located inside the cryostat and acquisition times of 60 s. The samples were cooled from room temperature down to $T_{c}=133$ K in a time of about 90 min. SERS signals were acquired in situ at decreasing $T_{c}$ = 294 K, 260 K, 230 K, 200 K, 170 K, 140 K, and 133 K temperatures. The temperature was stabilized at each of the above-reported $T_{c}$ values to permit the SERS measurements. Further SERS measurements were performed during the heating cycle at $T_{c}$ = 170 K, 230 K, 260 K, and 294 K, in a total time of about 90 min.

### 2.4. Data Analysis

The Raman and SERS spectra were normalized with respect to signal intensities by using the vector normalization method, i.e., the normalization of the signal to the standard deviation of points with respect to the average signal [27,28]. A best fit numerical procedure based on the Levenberg–Marquardt nonlinear least-square method (software routines by GRAMS/AI, 2001, Thermo Electron) was used for deconvoluting the spectra in term of Lorentzian functions to determine the main component modes and their parameters (i.e., the centers, widths, and intensities). Statistical analysis was performed by using one-way ANOVA test.

### 2.5. GbRS and SERS Advantages and Drawbacks

The joint use of GbRS and SERS opened a new interesting route for investigating the complex physio-chemical processes occurring in biological systems. GbRS and SERS can be particularly useful for studying the response of spores to stressing stimuli and improving the knowledge of underlying mechanisms [14,15,16,17,18,19,29]. In this work, we focused our attention on the spore secretions, which are strongly correlated to the spore state. SERS provided a sensible tool to assess fluid changes occurring around the spores when stimulated. The obtained information was complemented by GbRS that allowed for the estimation of the pH level of the secreted substances. Both based on Raman spectroscopy, the two methods share the advantages of the optical methods, such as a limited time-consumption, no need for complicated sample preparation procedures, high-sensitivity, and non-invasiveness. Information on the spore state is deduced from secretions, thus the interpretation of experimental results can be difficult to obtain in some cases. This can also be ascribed to the complex compositions of the secret substances, but the combined use of GbRS and SERS offers a unique tool for locally monitoring the microorganism evolution.

## 3. Results

**Figure 2:**
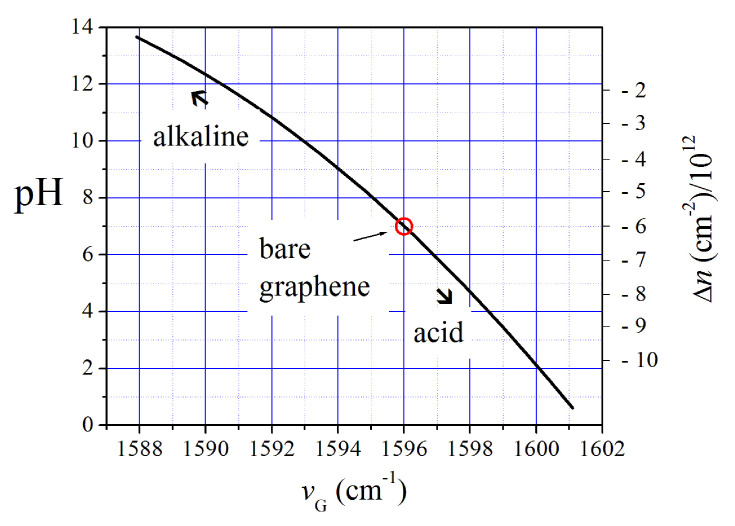
Expected dependence of pH and δn excess electronic doping on the $\nu_{G}$ energy of graphene.

Two main modes characterize the Raman spectrum of graphene: a sharp feature at $\nu_{G}\approx$ 1580 cm${}^{-1}$ (G mode) and a broad mode at $\nu_{2D}\approx 2650$ cm${}^{-1}$ (2D mode) [30]. Actually, the $\nu_{G}$ and $\nu_{2D}$ mode energies can differ from these values depending on the electronic doping of the graphene. Both hole- and electron-doping induce a shift of the G-mode energy to higher values that can be suitably estimated by Raman spectroscopy [12]. The acidity (pH level) of a fluid in contact with graphene surface influences the *n* electronic carrier density of this material and, consequently, its Raman spectrum. As said before, Paulus et al. developed a pH physiometer that works using this mechanism [11]. This method allows for the estimation of the pH level of a very small amount of substance. We applied it to monitor the pH changes of spore samples after the exposure to decreasing low temperatures, down to $T_{e}=100$ K. The dependence of pH and δn doping changes on the $\nu_{G}$ Raman G-mode energy of graphene was heuristically derived from preliminary calibrations [13] and is shown in Figure 2. The dependence of *n* on $\nu_{G}$ was extrapolated from the experimental data reported by Das et al. [12] and correlated to pH by calibration tests on known pH samples [13,31]. A linear correlation between pH and the excess doping δn with respect to the electron density of insulated graphene was derived:

(1) $$\mathrm{pH}=7.0+11.9\times 10^{12}\delta n$$

**Figure 3:**
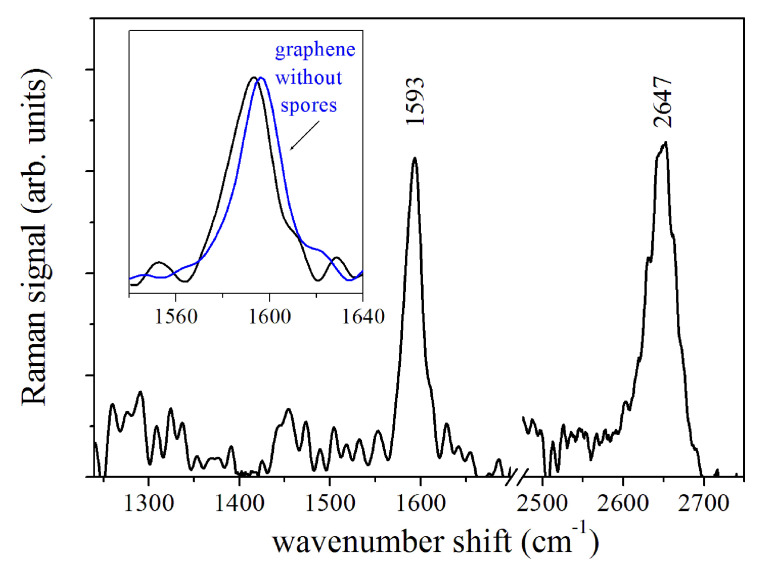
The Raman response of graphene. The $\nu_{G}$ mode region of the spectrum of graphene in contact with spores after the exposure to $T_{e}\;=\;200$ K is compared in the inset with the Raman response without spore (curve blue). The $\nu_{G}$ energy of the spectrum shifts to lower values, indicating an alkalinity increase (pH = 10.2±0.7) of the graphene with spores.

The reference Raman spectrum of graphene in contact with the spores suspension exposed to $T_{e}=200$ K is reported in Figure 3. The enlarged region of G-mode (black line) of this spectrum is shown in the inset and compared with the Raman spectrum acquired from graphene (blue line). A shift of $\nu_{G}$ to lower energy is clearly visible.

**Figure 4:**
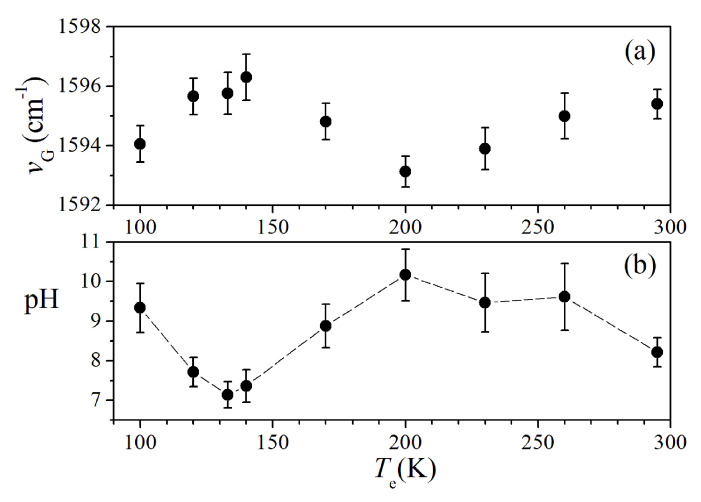
(**a**) Dependence of the graphene $\nu_{G}$ energy on the $T_{e}$ temperature. (**b**) Temperature dependence of pH of spore’s environment after low-temperature exposure. The pH values were determined from the value of $\nu_{G}$.

In order to correctly evaluate the $\nu_{G}$ shift, it is necessary to consider that the G-mode of the graphene placed on Si substrate (as in present case) is centered at $\nu_{G}=1596\pm 1\;$cm${}^{-1}$. This $\nu_{G}$ value is larger than the one expected for undoped graphene due to the occurrence of hole-doping processes caused by the contact with the Si substrate surface and air, as also reported by Paulus et al. [11]. The estimated $\delta n_{g}$ excess doping is of the order of $5.9\times 10^{12}$ cm${}^{-2}$. To take into account this initial doping, the evaluation of pH was done considering an effective doping density variation $\delta n^{*}\;=\;\delta n\;-\;\delta n_{g}$. The red-shift of the G-mode for the sample exposed to $T_{e}=200$ K (black curve in the inset of Figure 3) with respect to the reference spectrum of graphene indicates increased alkalinity of the spore sample (pH =10.2±0.7). The 2D-mode energy also changes towards a lower value, in agreement with the G-mode shift [11] (data not shown). Figure 4a resumes the results obtained by GbRS on the spore samples exposed to decreasing temperatures, namely, at $T_{e}=\;$294, 260, 230, 200, 170, 140, 133, 120, and 100 K. The pH values were estimated from these data and are reported in Figure 4b. At room temperature, the pH value is 8.2±0.4 slightly higher than the reference neutral value. After the exposure to temperatures varying between 260–200 K, pH increases up to 10.2±0.7. When the temperature was between 170–133 K, the pH decreases significantly with a minimum value equal to 7.1±0.3. The difference between the first two estimated pH levels is statistically significant (*p* < 0.025 level of significance) for the one-way ANOVA test.

**Figure 5:**
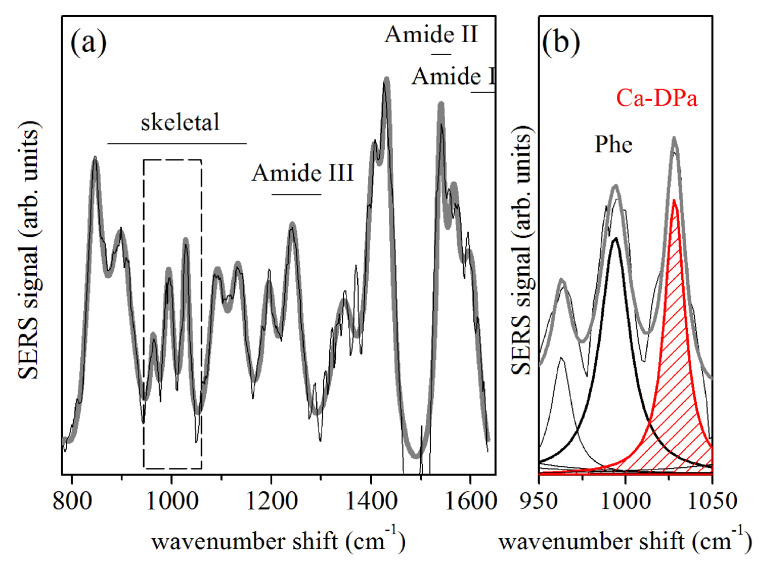
(**a**) Surface-Enhanced Raman Spectroscopy (SERS) response of spores of *Parageobacillus thermantarcticus* at room temperature. The spectrum was obtained by averaging 12 spectra acquired in different points of the sample. The experimental data was fitted by a convolution of Lorentzian components (gray curve). The framed area is reported magnified in (**b**). The peak with red dashed area was assigned to Ca${}^{2+}$-DPA.

**Figure 6:**
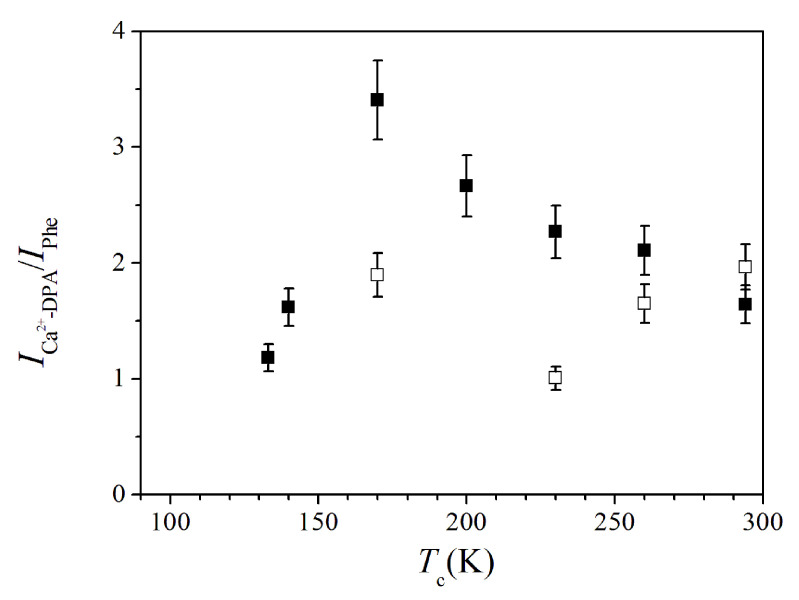
Temperature dependence of $I_{Ca^{2+}-DPA}$/$I_{phe}$ intensity ratio of spores after the cooling process at low-temperatures.

Interesting features were also obtained from the SERS response of spores suspensions exposed to low temperatures. Figure 5a shows the SERS response of a spore sample at room temperature. The shown spectrum was obtained by averaging 12 SERS spectra measured in different positions of the sample area. The gray curve indicates the fit of the experimental data by a convolution of Lorentzian peaks. In agreement with the literature, the bands related to the protein skeletal region (870–1150 cm${}^{-1}$ wavenumber) and the amide bands are individuated and indicated in Figure 5a [14,16,18]. The modes at about 810, 1024, 1405, and 1589 cm${}^{-1}$ were assigned to Ca${}^{2+}$-DPA [15,19]. Figure 5b provides an enlarged view of the 950–1050 cm${}^{-1}$ region. The Ca${}^{2+}$-DPA at about 1024 cm${}^{-1}$ is represented by the red dashed peak. The intensity of this mode is slightly larger than the one assigned to phenylalanine at ≈1000 cm${}^{-1}$ (from proteins), also reported in Figure 5b. The intensities of the modes at 1000 cm${}^{-1}$ and 1024 cm${}^{-1}$ were evaluated for all the SERS spectra acquired along the different cooling process. Figure 6 shows the temperature dependence of the $I_{Ca^{2+}-DPA}$/$I_{phe}$ ratio between the intensity value of the signals at 1024 cm${}^{-1}$ and 1000 cm${}^{-1}$ modes. Square black symbols refer to data collected during the cooling process, while empty squares indicate values obtained during the heating process. The $I_{Ca^{2+}-DPA}$/$I_{phe}$ ratio increased during the cooling cycle until it reached a maximum at $T_{c}=170$ K, after which it decreases. The $I_{Ca^{2+}-DPA}$/$I_{phe}$ ratio returned to the initial value of about 2 at room temperature, at the end of the temperature cycle.

## 4. Discussion

The observed pH changes in the spore suspensions after the exposure to low temperature can indicate the triggering of germination mechanisms. Figure 4a showed a slight increase in alkalinity from room temperature down to $T_{e}\approx 200$ K that indicates the release of proton accepting species like dipicolinate. A further lowering of the temperature down to $T_{e}=170$ K causes a pH decreases due to the release of H${}^{+}$ ions in the medium. The release of Ca${}^{2+}$-DPA and monovalent cations can be correlated to two steps of the initial stages of the germination process [2]. The onset of germination phenomena upon cooling to low temperature is supported by SERS. The SERS signals at 1024 and 1000 cm${}^{-1}$ are characteristic of Ca${}^{2+}$-DPA and the external coat proteins (Phe), respectively (see Figure 5). The $I_{Ca^{2+}-DPA}$/$I_{phe}$ intensity ratio is used as a marker of spore structure changes. Indeed, calcium dipicolinate is normally enclosed inside the inner core of the spore and an increase of $I_{Ca^{2+}-DPA}$/$I_{phe}$, reflects release processes through the external membrane. The $I_{Ca^{2+}-DPA}$/$I_{phe}\approx 1.5$ value was estimated by SERS for untreated spore samples. The $I_{Ca^{2+}-DPA}$/$I_{phe}$ ratio became larger when the temperature was decreased to $T_{c}\approx 170$ K, where a maximum value $I_{Ca^{2+}-DPA}$/$I_{phe}\approx 3.5$ was found. This behavior suggests that a release of Ca${}^{2+}$-DPA from the spore core occurred. In general, the release of Ca${}^{2+}$-DPA is activated by germination stimuli as the addition of nutrients or chemicals (like Ca${}^{2+}$-DPA itself, dodecylamine or peptidoglycan fragments) [7,9]. Application of stressor stimuli like pressure can also induce Ca${}^{2+}$-DPA release and a non-physiological germination pathway can take place [2,32,33]. These stimuli activate the SpoVA protein (stage V sporulation protein A) channels that are located in the inner membrane (Figure 1) which mediates the release of dipicolinate [2]. The increase of $I_{Ca^{2+}-DPA}$/$I_{phe}$ at $T_{c}\approx 170$ K indicates that a germination-like pathway is also induced by cooling processes. The further lowering of the temperature down to 133 K caused a rapid decrease of this ratio that can be explained assuming a relative increase of the $I_{phe}$ signal with respect to $I_{Ca^{2+}-DPA}$ caused by the swelling of the spores coat that usually occurs when the dipicolinate is released and the water (possibly present) can enter into the spore core. During the heating cycle, an increase at 170 K of the $I_{Ca^{2+}-DPA}$/$I_{phe}$ was found again, suggesting that at this temperature a major activation of SpoVA channels can take place. Nevertheless, when the room temperature is restored, the $I_{Ca^{2+}-DPA}$/$I_{phe}$ ratio returned to a value similar to the initial one, probably due to the end of the dipicolinate’s release. The temperature at which the germination process was induced ($T_{c}\approx 170$ K) is quite low, demonstrating the relevant resistance of *Parageobacillus thermantarcticus* spores to thermal excursions. This result is in agreement with previous studies on the effects of long-time storage at low temperatures (down to 148 K) that indicated a good resistance of spores in terms of survival level [34,35]. Below 170 K, the passage from spore to germinal cell state increases the vulnerability of the microorganism to the external agents and the probability of inactivation mechanisms onset decreasing their survival chances in hostile environments.

## 5. Conclusions

The results obtained here showed that the joint use of GbRS and SERS represents a valuable tool for monitoring the physio-chemical response of bacterial spores upon exposure to stressing stimuli as low temperatures. Indeed, thanks to the combination of these two techniques, it has been found that spores respond to cooling at low temperatures by undergoing a germination-like process. The SERS experiments evidenced that the cooling caused the dipicolinate release and the subsequent swelling of the spore due to the water intake in the core. These features are typical of the earlier germination stages. The graphene-based pH assessment confirmed this hypothesis proving the occurrence of monovalent cation release in concomitance with dipicolinate release. The reported study given an insight into the effects of low temperatures on spores states contributing to the knowledge of mechanisms controlling the life cycle of spores. GbRS and SERS could be usefully adopted for investigating the resistance of bacterial species to other stressing stimuli as heavy-ion irradiation or extreme conditions giving a relevant contribution to astrobiology. Furthermore, the results here reported can represent interesting issues in the biotechnological framework of food control and safety.
